# Occupational Burnout and Productivity Loss: A Cross-Sectional Study Among Academic University Staff

**DOI:** 10.3389/fpubh.2022.861674

**Published:** 2022-04-25

**Authors:** Shaimaa A. A. M. Amer, Sally Fawzy Elotla, Abeer Elsayed Ameen, Jaffer Shah, Ahmed Mahmoud Fouad

**Affiliations:** ^1^Department of Public Health, Occupational and Environmental Medicine, Faculty of Medicine, Suez Canal University, Ismailia, Egypt; ^2^Medical Research Center, Kateb University, Kabul, Afghanistan

**Keywords:** productivity, burnout, academic staff, Egypt, emotional exhaustion, occupational

## Abstract

**Background:**

Burnout has been endorsed with serious negative health- and work-related outcomes. This study is aimed to assess the prevalence of burnout and its association with work productivity among academic staff.

**Methods:**

This cross-sectional study involved 240 academic staff working at a public university in Egypt. Participants were invited to complete a web-based questionnaire involving basic personal, health, and work-related characteristics. Besides, Maslach Burnout Inventory-Human Services Survey (MBI-HSS) was used to assess occupational burnout dimensions (i.e., emotional exhaustion “EE,” depersonalization “DP,” and personal accomplishment “PA”), while work productivity was assessed with the Health and Work Performance Questionnaire (HPQ).

**Results:**

In total, 28% of respondents scored high in EE [95% confidence interval (CI): 22.5–33.8%], 18.3% high in DP (95% CI: 13.8–3.6%), and 88.3% scored low in PA (95% CI: 83.8–91.9%). Seventy percent of respondents scored high in only one burnout dimension, 21.7% scored high in two dimensions, while 7.1% scored high in all three dimensions. Multivariable analysis showed that EE was the only burnout dimension that showed a statistically significant association between absenteeism and presenteeism rates. The absenteeism rates among respondents with moderate and high EE were 2.1 and 3.3 times the rates among those with low EE, respectively. Likewise, the presenteeism rates among respondents with moderate and high EE were 2.4 and 4.7 times the rates among those with low EE, respectively.

**Conclusions:**

Academic staff showed a high prevalence of at least one burnout dimension. Moderate and high EE scores were significantly associated with increased productivity loss when compared to low EE.

## Introduction

The world health organization (WHO), in its latest revision of the International Classification of Diseases (ICD-11, 2019), has officially classified burnout as an occupational phenomenon that should not apply to describe experiences in other areas of life. Building on previous Maslach's work, burnout was defined in the WHO's ICD-11 as “a syndrome conceptualized as resulting from chronic workplace stress that has not been successfully managed and characterized by three dimensions: feelings of energy depletion or exhaustion; increased mental distance from one's job or feelings of negativism or cynicism related to one's job; and reduced professional efficacy” (1, 2).

Burnout has been addressed in various occupational settings, such as universities, with serious negative implications on job retention, commitment, satisfaction, and performance (3). Burnout among academic staff impairs not only their mental and physical health but also reduces their perceived self-efficacy and responsiveness to students' needs, leading to reduced effectiveness of the educational process and students' satisfaction (4–6).

Academic institutions have been recognized for the increasingly demanding work environments and high levels of stress that have significantly increased over the last few decades (7–10). Academic staff is strained by a variety of challenging and often conflicting roles, such as providing high-quality teaching and supervision to a growing number of students, publishing innovative research in high-impact journals, application of research grants, sustaining managerial and entrepreneurial skills, and tenure-related accomplishment (8, 9, 11). Coping with these complex work demands, increasing role-ambiguity and role-conflicts, job insecurity, and competitiveness, is very stressful and has been associated with the increased psychological strain, depletion of mental resources, and burnout among academic staff (7, 12, 13).

The burden of burnout among academic staff has recently received increasing attention because of the growing global changing in curricula design and technology. Digital transformation, expansion of teleworking, and dynamic multi-task duties have created further pressure on meeting the high-performance expectations in universities, particularly, in public universities (11, 14).

Egypt is currently seeking to improve its educational system's quality to conform to international systems along with its national strategy for sustainable development, Egypt vision 2030. With the expansion of the private universities in Egypt, competition between the higher education institutions became more severe. As a competitive advantage, faculty performance undergoes continuous assessment and monitoring by the human resources departments (15). Besides, as a requirement for their promotion, faculty members in Egypt are overwhelmed with scholar productivity, particularly, the international peer-reviewed publication, which is also required to increase the ranking of their universities. In addition, academic staff in Egypt has to be involved in community outreach programs and projects that emphasize the community needs, ambitions, and the market (16). The tenure track is the only professor's pathway to the promotion and academic job security in all Egyptian public universities. Professor's contributions in three areas, i.e., research, teaching, and service to the university and community, undergo vigorous evaluation every at least 5 years. However, private universities have mixed systems that are mostly non-tenure track, which depends on temporary contracts renewed annually according to staff performance and resources.

Burnout has been identified as a disruptive factor in organizational performance and costs (17). Physical and mental health significantly moderate the relationship between work-related factors and individual work productivity (18). Loss of work productivity involves increased absenteeism, presenteeism, and reduced work ability (18, 19). Although work productivity has been widely studied in different physical and mental health conditions and disabilities (20–22), few studies have addressed the association with burnout.

Studying the productivity of academic staff in Egypt is necessary, given their role in Egypt's economic and social transformation as described in Egypt's strategy for sustainable development, Egypt vision 2030. Escalating their productivity is essential for their institutions' development and sustainability. Occupational stress and burnout are major challenges for the individual and institutional productivity and turnover, representing 12% of all absent workforce (23). Furthermore, increased productivity loss and turnover have many economic implications that could boost Egypt's struggle for the economic transformation and social welfare. Therefore, the purpose of this study was to assess the prevalence of burnout and its association with work productivity in a sample of academic staff in Egypt.

## Methods

This cross-sectional study involved academic staff who had worked for at least 6 months at Suez Canal University, Ismailia, Egypt (a public university in Egypt). We obtained ethical approval from the IRB of the faculty of medicine at xxx university. Further permissions to conduct this study were taken from the appropriate authorities at the faculties of art and social sciences, life sciences, and other sciences. The information technology and human resource departments were approached to get the email addresses of eligible staff. An invitation that includes information about the purpose and procedures of the study was emailed to a convenient sample of 240 academic staff across the faculties (proportionally stratified by the specialties: Art and Social Sciences, Life Sciences, and Other Sciences). The size of this sample (i.e., 240) was calculated using Epi-Info^®^ StatCalc software, version 7.2.4.0 (Centers for Disease Control and Prevention, Atlanta, GA, USA), giving an expected percentage of 38.9% of participants who had a high burnout and decreased work productivity (24), 95% level of confidence, 6.5% absolute precision level, and the addition of a 10% of the calculated number to compensate for dropout.

In the emailed invitation, respondents were asked if they would give their consent to take part in this study. Respondents who gave their consent to take part in this study were asked to click a link for a web-based questionnaire. The questionnaire included basic data about the socio-demographic characteristics (e.g., age, residence, marital status, and education); lifestyle behaviors (e.g., smoking, physical activity, and alcohol consumption); self-reported body weight and height; and work characteristics (e.g., job categories, duration of employment, and work patterns). Regular physical activity was defined according to the World Health Organization (WHO) as “at least 150–300 min of moderate-intensity aerobic physical activity; at least 75–150 min of vigorous-intensity aerobic physical activity; or an equivalent combination of moderate- and vigorous-intensity activity throughout the week ≥ 150 min/week” (25). Body mass index (BMI) was calculated as the body weight in kg divided by the squared root of the height in cm. BMI values ≥ 30 kg/m^2^ denote obesity, while BMI values of 25–29.9 were overweight (26).

Maslach Burnout Inventory-Human Services Survey (MBI-HSS) was used to assess occupational burnout on three subscales: emotional exhaustion (EE) (9 items), depersonalization (DP) (5 items), and professional accomplishment (PA) (8 items), with a 7-point Likert scale (0 = never, 1 = a few times a year or less, 2 = once a month or less, 3 = a few times a month, 4 = once a week, 5 = a few times a week, and 6 = every day). High levels of EE and DP subscales and low levels of PA suggest burnout (27). The Arabic version of MBI-HSS was validated in many Arab populations (28–31) and authors have reported satisfactory psychometric properties of reliability and validity. The Arabic translation of MBI-HSS was adapted to the vernacular of Egyptian by a panel of experts who performed forward-backward translation according to the WHO guidelines for translation and validation of questionnaires. A pilot testing was performed following a minor revision of dialects. The internal consistency of the Arabic MBI-HSS in our sample was satisfactory, with Cronbach's alfa of 0.78, 0.81, and 0.72 for EE, DP, and PA, respectively.

This study made the use of the WHO's Health and Work Performance Questionnaire (HPQ) to assess work productivity among respondents. The Arabic translation of the HPQ was performed in an earlier work by the Fouad et al. (21) who translated it using forward-backward translation according to the WHO guidelines for translation and validation of questionnaires. In HPQ, the assessment of work productivity relies on measuring absenteeism and presenteeism in days through a series of self-administered questions as described by Kessler et al. (32).

### Statistical Analysis

All data manipulation and statistical analyses were performed with SPSS Software version 25 (IBM Corporation, Armonk, NY, USA). Categorical variables were presented as frequencies and percentages (%) while continuous and discrete data were presented as mean ± standard deviation (SD). Normality of continuous data [e.g., burnout domains' scores (EE, DP, and PA), absenteeism, and presenteeism variables] was tested by the Kolmogorov-Smirnov test. The median and interquartile ranges were used to summarize variables, which were not normally distributed. Associations between categorical variables were tested for statistical analysis using a chi-square test or Fisher's exact test (if > 20% of expected values were <5). Mann-Whitney and Kruskal-Wallis tests were used to test for the statistical significance of differences in no-normally distributed variables across the categories of other study variables. Spearman's correlation was used for testing the significance of bivariate associations.

Multivariable analyses were performed using negative binomial regression models because our dependent variables (i.e., absenteeism and presenteeism) were counted data with many zeros measured in a 28-day time frame and because of the over-dispersion of these variables in our sample. Rate ratios and their 95% confidence intervals (CIs) were reported for each model. Values of *p* < 0.05 was considered statistically significant.

## Results

This study involved 240 academic staff; 73% were women and 27% were men, with an average age of 42.5 (±7.9) years. A detailed description of the demographic, health, and work characteristics of the studied sample is summarized in Table 1. Twenty-eight percentage of respondents scored high for EE (95% CI: 22.5–33.8%), 18.3% scored high for DP (95% CI: 13.8–23.6%), while 88.3% scored low for PA (95% CI: 83.8–91.9%). Over two-thirds of respondents scored high for burnout in only one dimension (70.0%, 95% CI: 64.0–75.5%), 21.7% scored high for burnout in two dimensions (95% CI: 16.8–27.2%), and 7.1% scored high for burnout in all three dimensions (95% CI: 14.3–10.9%). Only three respondents (1.3%, 95% CI: 0.4–3.3%) did not score high for burnout in any dimension.

**Table 1:** Distribution of respondents' burnout scores by their demographic, health, and work characteristics (*N* = 240).

**Characteristics**	***n* (%)**	**Occupational burnout domains, median (IQR)**					
		**EE**	***p*-value**	**DP**	***p*-value**	**PA**	***p*-value**
**Age, years**
<40	89 (37.1%)	23.0 (15.0, 30.0)	0.11	11.0 (9.0, 12.0)	0.098	26.0 (21.0, 29.0)	0.576
40–49	104 (43.3%)	21.0 (13.5, 29.0)		11.0 (8.0, 13.0)		26.0 (20.0, 29.0)	
50 or older	47 (19.6%)	17.0 (12.0, 25.0)		10.0 (7.0, 12.0)		25.0 (21.0, 29.0)	
**Gender**
Male	175 (72.9%)	18.0 (13.0, 28.0)	0.005^*^	11.0 (8.0, 12.0)	0.668	27.0 (21.0, 29.0)	0.164
Female	65 (27.1%)	24.0 (18.0, 30.0)		11.0 (8.0, 12.0)		24.0 (18.0, 30.0)	
**Residence**
The same governorate	197 (82.1%)	18.0 (13.0, 26.0)	0.001^*^	11.0 (8.0, 12.0)	0.272	26.0 (21.0, 29.0)	0.309
Remote governorate	43 (17.9%)	25.0 (20.0, 33.0)		11.0 (8.0, 13.0)		24.0 (17.0, 28.0)	
**Marital status**
Single	11 (4.6%)	31.0 (19.0, 34.0)	0.054	12.0 (10.0, 14.0)	0.287	22.0 (20.0, 30.0)	0.46
Married	214 (89.2%)	20.0 (13.0, 29.0)		11.0 (8.0, 12.0)		26.0 (21.0, 29.0)	
Divorced or widowed	15 (6.3%)	18.0 (13.0, 23.0)		10.0 (8.0, 12.0)		27.0 (24.0, 30.0)	
**Number of offspring**
None	33 (13.8%)	21.0 (16.0, 31.0)	0.283	12.0 (10.0, 12.0)	0.155	28.0 (22.0, 30.0)	0.23
1–2	64 (26.7%)	23.0 (12.0, 30.0)		11.0 (8.5, 12.0)		26.0 (20.0, 29.0)	
3 or more	143 (59.6%)	19.0 (14.0, 28.0)		11.0 (8.0, 12.0)		26.0 (21.0, 29.0)	
**Age of youngest offspring (*n* = 207)**
<5	85 (41.1%)	21.0 (13.0, 29.0)	0.852	11.0 (8.0, 12.0)	0.976	25.0 (19.5, 29.0)	0.46
5–17	106 (51.2%)	20.5 (15.0, 27.0)		11.0 (8.0, 12.0)		25.0 (20.0, 29.0)	
18+	16 (7.7%)	18.0 (12.0, 30.0)		11.0 (8.0, 12.0)		27.0 (21.0, 29.0)	
**Body mass index class**
Normal	42 (17.6%)	21.0 (13.0, 30.0)	0.633	11.5 (9.0, 12.0)	0.751	26.5 (22.0, 30.0)	0.542
Overweight	110 (46.0%)	19.0 (14.0, 26.0)		11.0 (8.0, 12.0)		25.0 (21.0, 29.0)	
Obese	87 (36.4%)	22.0 (14.0, 29.0)		11.0 (8.0, 12.0)		26.0 (19.0, 30.0)	
**Cigarette smoker**
Never	210 (87.5%)	20.5 (14.0, 29.0)	0.71	11.0 (8.0, 12.0)	0.642	26.0 (21.0, 29.0)	0.53
Yes	30 (12.5%)	18.0 (12.0, 29.0)		10.5 (8.0, 12.0)		26.5 (17.0, 29.0)	
Cig. smoking pack.year	5.6 (2.2, 20.0)^a^	−0.210^b^	0.266	−0.044^b^	0.816	0.034^b^	0.857
**Regular physical activity**
No	212 (88.3%)	21.0 (14.0, 29.0)	0.132	11.0 (8.0, 12.0)	0.29	26.0 (20.0, 29.0)	0.946
Yes	28 (11.7%)	16.5 (12.0, 27.0)		12.0 (9.5, 12.0)		26.0 (22.0, 29.0)	
**Chronic diseases**
None	172 (71.7%)	19.5 (14.5, 27.5)	0.64	11.0 (8.0, 12.0)	0.371	26.0 (20.0, 29.0)	0.145
Single disease	53 (22.1%)	24.0 (12.0, 31.0)		11.0 (9.0, 12.0)		27.0 (21.0, 30.0)	
Multiple diseases	15 (6.3%)	19.0 16.0, 35.0)		10.0 (8.0, 14.0)		27.0 (21.0, 37.0)	
**Current job title**
Lecturer	133 (55.4%)	22.0 (15.0, 30.0)	0.223	11.0 (9.0, 12.0)	0.515	26.0 (21.0, 29.0)	0.803
Associate Professor	62 (25.8%)	18.0 (14.0, 26.0)		11.0 (8.0, 12.0)		26.5 (20.0, 30.0)	
Professor/Professor Emeritus	45 (18.8%)	18.0 (12.0, 25.0)		10.0 (8.0, 12.0)		26.0 (23.0, 29.0)	
**Professional specialties**
Art and Social Sciences	66 (27.5%)	18.5 (13.0, 27.0)	0.015^*^	11.0 (9.0, 12.0)	0.784	26.5 (22.0, 29.0)	0.039^*^
Life Sciences	60 (25.0%)	24.0 (17.5, 32.5)		11.0 (8.0, 13.0)		24.0 (16.5, 28.5)	
Other Sciences	114 (47.5%)	18.0 (13.0, 25.0)		11.0 (8.0, 12.0)		27.0 (21.0, 29.0)	
**Years of employment**
<10	51 (21.3%)	21.0 (14.0, 29.0)	0.115	11.0 (10.0, 12.0)	0.283	26.0 (21.0, 30.0)	0.74
10–20	123 (51.2%)	22.0 (15.0, 30.0)		11.0 (8.0, 12.0)		26.0 (20.0, 29.0)	
More than 20	66 (27.5%)	17.5 (12.0, 25.0)		11.0 (7.0, 12.0)		26.0 (21.0, 29.0)	
**Workdays/week**	4 (4, 5)^a^	0.088^b^	0.172	0.172^b^	0.007^*^	0.179^b^	0.005^*^
**Workhours /day**	6 (5, 8)^a^	−0.041^b^	0.531	0.041^b^	0.524	0.111^b^	0.086
**Telework hours/week**	16 (10, 20)^a^	0.138^b^	0.033^*^	0.131^b^	0.044^*^	0.167^b^	0.010^*^

a *Median (interquartile range).*

b *Spearman's rho correlation coefficient.*

* *Statistically significant value of p (< 0.05); Mann-Whitney or Kruskal-Wallis tests.*

*EE, emotional exhaustion; DP, depersonalization; PA, professional accomplishment; IQR, interquartile range*.

Table 1 shows the distribution of respondents' scores in the different burnout dimensions by their demographic, health, and work characteristics. Women, remote residence, life science specialties, and increasing telework hours per week were significantly associated with higher median scores for burnout in the EE dimension. Increased number of workdays and telework hours per week were significantly associated with higher median scores for burnout in the DP dimension. Life science specialties were associated with lower median score for burnout in PA dimension, while increasing the number of workdays and telework hours per week was associated with higher median scores for PA.

During the last 28 days, respondents reported average absenteeism days of 1 day (range: 0–4 days) and average presenteeism days of 2.7 days (range: 0–9.3 days). Table 2 shows the distribution of respondents' median absenteeism and presenteeism days by their demographic, health, and work characteristics. Absenteeism was significantly associated with only the professional specialties; respondents from life sciences specialties showed significantly higher median absenteeism days than other specialties. Presenteeism was significantly associated with respondents' residence, professional specialties, workdays per week, and telework hours per week. The median presenteeism days were significantly high among respondents who were living in remote governorates and working in life sciences specialties. The number of workdays per week and telework hours per week showed significant positive, but weak, correlation with the number of presenteeism days.

**Table 2:** Distribution of respondents' absenteeism and presenteeism days by their demographic, health, and work characteristics (*N* = 240).

**Characteristics**	***n***	**Productivity loss (days), median (IQR)**			
		**Absenteeism**	***p*-value**	**Presenteeism**	***p*-value**
**Age, years**
<40	89	0.0 (0.0, 1.5)	0.737	2.4 (1.0, 4.0)	0.204
40–49	104	0.0 (0.0, 1.5)		2.6 (1.4, 4.3)	
50 or older	47	0.0 (0.0, 1.5)		2.0 (1.0, 3.2)	
**Gender**
Male	175	0.0 (0.0, 1.5)	0.247	2.0 (1.0, 4.0)	0.08
Female	65	0.5 (0.0, 2.0)		3.0 (1.5, 4.0)	
**Residence**
The same governorate	197	0.0 (0.0, 1.5)	0.109	2.1 (1.0, 4.0)	0.012^*^
Remote governorate	43	1.0 (0.0, 2.0)		3.2 (1.5, 5.0)	
**Marital status**
Single	11	1.5 (0.0, 2.6)	0.446	3.0 (1.8, 4.5)	0.845
Married	214	0.0 (0.0, 1.5)		2.3 (1.0, 4.0)	
Divorced or widowed	15	0.5 (0.0, 1.5)		1.8 (1.0, 3.9)	
**Number of offspring**
None	33	0.0 (0.0, 2.0)	0.709	2.4 (1.0, 3.5)	0.537
1–2	64	0.0 (0.0, 1.5)		2.5 (1.3, 4.0)	
3 or more	143	0.0 (0.0, 1.5)		2.4 (1.2, 4.0)	
**Age of youngest offspring (*n* = 207)**
<5	85	0.5 (0.0, 2.0)	0.74	3.1 (1.2, 5.3)	0.571
5–17	106	0.0 (0.0, 1.5)		2.5 (1.3, 4.0)	
18+	16	0.0 (0.0, 1.5)		2.1 (1.2, 4.0)	
**Body mass index class**
Normal	42	0.0 (0.0, 2.0)	0.852	2.1 (1.0, 4.0)	0.806
Overweight	110	0.3 (0.0, 1.5)		2.4 (1.4, 4.0)	
Obese	87	0.0 (0.0, 1.5)		2.4 (1.0, 4.0)	
**Cigarette smoker**
Never	210	0.0 (0.0, 1.5)	0.318	2.5 (1.2, 4.0)	0.293
Yes	30	0.0 (0.0, 1.0)		2.0 (1.0, 3.2)	
**Cig. smoking pack.year**	240	0.125^a^	0.509	0.078^a^	0.681
**Regular physical activity**
No	212	0.0 (0.0, 1.5)	0.208	2.5 (1.2, 4.0)	0.223
Yes	28	0.0 (0.0, 1.0)		2.0 (1.0, 3.5)	
**Chronic diseases**
None	172	0.0 (0.0, 1.5)	0.453	2.4 (1.2, 3.9)	0.315
Single disease	53	0.5 (0.0, 1.5)		2.8 (0.8, 5.0)	
Multiple diseases	15	1.0 (0.0, 2.0)		2.5 (1.3, 5.0)	
**Current job title**
Lecturer	133	0.0 (0.0, 1.5)	0.591	2.5 (1.2, 4.0)	0.7
Associate Professor	62	0.0 (0.0, 1.5)		2.9 (1.0, 4.0)	
Professor/Professor Emeritus	45	0.5 (0.0, 1.0)		2.0 (1.2, 3.0)	
**Professional specialties**
Art and Social Sciences	66	0.0 (0.0, 1.0)	0.040^*^	2.4 (1.0, 4.0)	0.039^*^
Life Sciences	60	0.8 (0.0, 2.0)		3.1 (1.4, 4.9)	
Other Sciences	114	0.0 (0.0, 1.5)		2.0 (1.0, 3.5)	
**Years of employment**
<10	51	0.5 (0.0, 1.5)	0.901	2.2 (1.2, 4.0)	0.581
10–20	123	0.0 (0.0, 1.5)		2.6 (1.0, 40)	
More than 20	66	0.0 (0.0, 1.5)		2.0 (1.1, 3.5)	
**Workdays/week**	240	0.038^a^	0.561	0.140^a^	0.031^*^
**Workhours/day**	240	0.031^a^	0.631	−0.016^a^	0.809
**Telework hours/week**	240	−0.051^a^	0.434	0.136^a^	0.037^*^

*IQR, interquartile range.*

a *Spearman's rho correlation coefficient.*

* *Statistically significant value of p (< 0.05); Kruskal-Wallis test*.

Table 3 shows the adjusted associations between the level (low, moderate, and high) of respondents' scores on each burnout dimension (EE, DP, and PA) and the absenteeism and presenteeism rates in the last 28 days. The EE was the only burnout dimension that showed a statistically significant association between absenteeism and presenteeism. Respondents with moderate and high burnout scores in the EE dimension had a significantly high absenteeism rate; 2.1 and 3.3 times the rates among respondents with low burnout scores in EE, respectively. Likewise, respondents with moderate and high burnout scores in the EE dimension had a significantly high presenteeism rate; 2.4 and 4.7 times the rates among respondents with low burnout scores in EE, respectively. All of these associations were adjusted for respondents' age, gender, residence, marital status, number of children, professional specialties, years of employment, workdays per week, telework hours/week, work hours per day, regular physical activity, and number of chronic diseases.

**Table 3:** Multivariable analysis^a^ for the association between burnout and productivity loss (absenteeism and presenteeism rates) in the studied sample (*N* = 240).

**Burnout domains**	***n***	**Absenteeism (days)**			**Presenteeism (days)**		
		**Rate ratio**	**95% CI**	***p*-value**	**Rate Ratio**	**95% CI**	***p*-value**
**Emotional exhaustion**
Low	85	1			1		
Moderate	88	2.05	1.05–4.01	0.036^*^	2.38	1.32–4.29	0.004^*^
High	67	3.33	1.43–7.79	0.005^*^	4.67	1.97–11.05	0.000^*^
**Depersonalization**
Low	40	1			1		
Moderate	156	1.01	0.46–2.25	0.973	1.29	0.66–2.53	0.465
High	44	1.47	0.48–4.52	0.497	1.14	0.37–3.49	0.826
**Personal accomplishment**
High	4	1			1		
Moderate	24	0.38	0.06–2.36	0.297	1.43	0.30–6.79	0.650
Low	212	0.59	0.11–3.28	0.550	1.37	0.28–6.78	0.700
Intercept	240	0.24	0.01–10.9	0.466	0.11	0.004–3.49	0.212

*CI, confidence interval.*

a *Negative binomial regression model; adjusted for age (years), gender, residence, marital status, number of children, professional specialties, years of employment, workdays per week, telework hours/week, work hours per day, regular physical activity, and number of chronic diseases.*

* *Statistically significant at 95% level of confidence*.

## Discussion

Although burnout has been extensively described among health professionals worldwide and in the Eastern Mediterranean region (33), limited research was performed among academic university staff, particularly in low middle-income countries. In developed countries, burnout among faculty has been an important issue for decades with increasing responsibilities and tasks diversity (34). In the current study, 28 and 18.3% of the academic staff had high EE and DP, respectively, while 88.3% had low PA. Seventy percent of respondents reported a high burnout in at least one dimension while 7.1% reported high burnout in all dimensions. The EE was significantly associated with productivity loss (i.e., days of absenteeism and presenteeism). A Supplementary Table S1 summarizes our study findings when compared to similar studies in the literature.

The prevalence of burnout in our study is nearly consistent with a study conducted by Aleves et al. (35), which reported that 33.6% of a sample of 366 faculty members in a public Brazilian university reported high burnout. Other studies in Europe, North and South America reported variable estimates of burnout prevalence among faculty members, ranging from 14 to 40% (36–38).

In Soler et al. (24), a multinational study was conducted on 1,393 family physicians in 12 European Countries (Bulgaria, Croatia, France, Greece, Hungary, Italy, Malta Poland, Spain, Sweden, Turkey, and United Kingdom), 43.0% of respondents scored high on EE burnout dimension while 35.3 and 32.0% reported high DP and PA, respectively. Compared to Soler's findings, our study's respondents scored low on EE and DP burnout domains while they scored very high on the PA domain.

The current study showed that the female gender was associated significantly with higher EE scores. This finding was consistent with the Alves et al.'s (35) study, which reported that women were more exhausted than men. In contrast, Soler et al. (24) reported that men had significantly higher EE scores than women.

The dominance of men working in academic jobs at the Egyptian universities may explain the gender difference in our study (39). Despite the paucity of national estimates for female academic staff in the Egyptian universities, findings from a German Academic Exchange Service (DAAD)-funded project entitled “Gender Equality in the Egyptian Higher Education System, carried out from 2012 to 2014” showed a low frequency of female academic staff in the Egyptian universities (ranged from 34.6 to 41.9%), particularly at universities in Upper Egypt (ranged from 5.9 to 18.5%). Furthermore, the project's findings denoted that most Egyptian universities lack programs, which maintain work-life balance and facilitate the integration of female graduates into the academic careers (40).

The current study's findings were consistent with an earlier report by Miller (41) in that living in remote areas was associated with higher EE among teachers than in living in the same governorate where they work, given that individuals who travel long distances for work were more susceptible to bad weather and other road and time obstacles than those who live in areas of a short distance from work.

Our study showed that faculty members in health and life sciences specialties had significantly higher EE and less PA. These results were not consistent with Alves et al. (35), which reported that there were no significant differences among faculty members from the different fields of knowledge. Furthermore, our study results indicated that increasing the number of telework hours per week was significantly associated with increased EE, DP, and PA. Telework implies remote work where the online work is an important component. Whenever the academic institutions adopt a remote teaching model, they have to invest more in the correct technologies and IT support. Using the wrong technology increases the burden on the faculty members to make the remote teaching model successfully work, which is basically a recipe for increasing workload and work-family conflict for burnout (42).

Female academic staff in our study experienced more productivity loss (i.e., higher median days of absenteeism and presenteeism) than male staff. These findings agreed with Miller's study (41). This finding could be explained by the increased work-family conflict and the higher burnout among female academic staff. Female faculty members combine more work and home responsibilities than men. A study by Burk and El-Kot (43) among Egyptian professionals has reported higher levels of work-family conflict, exhaustion, and psychosomatic symptoms among working women as compared to men. Another study by Marafi (44) reported that the main challenge and the leading cause of work-life conflict among professional female workers in Egypt was the lack of time available for women to fulfill their work duties and family responsibilities.

The current study results showed that moderate and high emotional exhaustion were significant predictors of the increased absenteeism and presenteeism. These findings were consistent with the findings of a Chinese study by Pie et al. (45) in which respondents with a medium or high EE and PD had twice or more presenteeism than those with low EE or PD. Furthermore, our findings were consistent with a systematic review by Dewa et al. (19), which reported a significant negative relationship between burnout and productivity which were assessed in four domains: the number of sick leave days, intent to continue practicing, intent to change jobs, and work ability. Likewise, Ruitenburg et al. (46), a study from one academic medical center in the Netherlands, found that physicians' self-perceived insufficient work ability was associated with high Burnout. High scores in EE and DP dimensions of burnout had significantly 9.5 times greater odds of having self-perceived insufficient work ability. In contrast, our study showed a similar association but of less magnitude and with only the EE domain. In a study by Woo et al. (34) on the relationship between faculty burnout and scholarly productivity among 251 faculty members in the U.S. results, burnout was a predictive of scholarly productivity (r = – 0.216; *p* < 0.05). Faculty members with a high burnout showed a significantly low scholarly productivity as compared to those who exhibited less burnout. Soler et al. (24) reported an increased sick leave with different high burnout dimensions: high EE and DP and with Low PA. In contrast, the study of Siu et al. (47) showed no relation between different burnout dimensions and sick leaves.

In the current study, absenteeism was one of the outcomes of burnout and a cause of productivity loss as absent teachers are typically replaced by less qualified substitutes or their tasks were redistributed to their colleagues, which leads to an increase in the burden on the rest of the colleagues and thus the efficiency, instructional intensity and consistency, and quality of education may decline. Moreover, burnout syndrome can also incapacitate faculty members from work through different personal dysfunctions, such as serious psychological and physical disorders (i.e., presenteeism) (48). Findings of our study contribute significantly to the literature on burnout among academic staff in universities and have several practical implications. University policies should be implemented at the individual and institutional levels to mitigate burnout and foster productivity among academic staff. Such arrangements should be particularly targeted at female faculty, working in life-sciences specialties, increasing telework, and accommodation.

The main limitations in this study that should be taken into consideration while interpreting its findings were the single-center experience (i.e., single academic institution) and the cross-sectional design, which cannot ascertain the causal inferences. For future research, other methodological designs can be used (with longitudinal studies and probabilistic sampling) by including universities of different legal natures (public and private) and also including more detailed specialties of academia. Quantification of the magnitude of association between burnout and productivity loss should be evaluated in further studies.

## Conclusions

Academic staff showed a high prevalence of at least one burnout dimension. Moderate and high burnout scores in the EE dimension were significantly associated with high productivity loss as higher absenteeism and presenteeism rates when compared to low EE. Women, remote residence, life science specialties, and increasing telework hours per week were significantly associated with higher median scores for burnout in the EE dimension. Increased number of workdays and telework hours per week was significantly associated with higher median scores for burnout in the DP dimension. Life science specialties were associated with lower median score for burnout in the PA dimension, while increasing the number of workdays and telework hours per week were associated with higher median scores for PA.

## Data Availability Statement

The raw data supporting the conclusions of this article will be made available by the authors, without undue reservation.

## Ethics Statement

This study was approved by the Ethical Committee at Faculty of Medicine, Suez Canal University, Ismailia, Egypt. All participants gave their informed consent prior to participation in this study.

## Author Contributions

AF, SE, and SA: conceived the idea and designed the study. SA, SE, and AA: collected the data. AF, SA, and SE: analyzed and interpreted the data. AF, SA, SE, AA, and JS: wrote the manuscript. All authors reviewed and approved this version of the manuscript.

## Conflict of Interest

The authors declare that the research was conducted in the absence of any commercial or financial relationships that could be construed as a potential conflict of interest.

## Publisher's Note

All claims expressed in this article are solely those of the authors and do not necessarily represent those of their affiliated organizations, or those of the publisher, the editors and the reviewers. Any product that may be evaluated in this article, or claim that may be made by its manufacturer, is not guaranteed or endorsed by the publisher.
