# Supplementary material for: Occupational Burnout and Productivity Loss: A Cross-Sectional Study Among Academic University Staff
Source: Front Public Health. 2022 Apr 25;10:861674. doi: 10.3389/fpubh.2022.861674 (PMC9082414; doi:10.3389/fpubh.2022.861674)
Supplement: Supplementary file 1 [file Table_1.docx]

**Supplementary Table 1 (S1). Comparison of the study findings with similar studies in the literature**

| **Studies** | **Year** | **Country** | **Occupational group** | **Study Design** | **Burnout Instrument** | **Sample size** | **Main Findings** | **Current study's findings** |
| --- | --- | --- | --- | --- | --- | --- | --- | --- |
| **Pei** et al. | 2020 | China | Physicians | Cross-sectional study | 15-item Maslach's Burnout Service Inventory (MBI-GS) | 1376 | Moderate job burnout: 86.8%  Severe job burnout: 6.0% | High burnout***:*** 7.1% (***Higher***). |
| **Alves** et al. | 2019 | Brazil | Faculty members | Cross-sectional study | Oldenburg Burnout Inventory–OLBI | 366 | High burnout: 36.6%.  Women more exhausted than men. | High burnout***:*** 7.1% (***Lower***).  Women more exhausted than men (***Similar***). |
| **Woo** et al. | 2017 | U.S | Faculty members | Cross-sectional study | Copenhagen Burnout Inventory (CBI) | 251 | High burnout predict low scholarly productivity. | High EE predict productivity loss (***Similar***) |
| **Dewa** et al. | 2014 | US, China, Hong Kong, Europe | Physicians | Systematic review | MBI-HSS | 3885 | Burnout is associated with decreased productivity and increased intention to leave a position or the field. | High EE predict productivity loss (***Similar***) |
| **Siu** et al. | 2012 | Hong Kong | Physicians | Cross-sectional study | MBI-HSS | 226 | High burnout: 31.4%  Mean Scores: EE = 27.2, DP = 10.9, PA = 31.6  Median Sick Leave Days in the Last Year by Burnout: High burnout = 1, Non-high burnout = 0.25 (p-value >0.05) | High burnout***:*** 7.1% (***Lower***).  Moderate and high EE significantly predict high absenteeism rate (2.1 and 3.3 times the rates if low EE, respectively) (**significantly** ***higher rate of absenteeism***) |
| **Ruitenburg** et al. | 2012 | Netherlands | Physicians in one academic medical center | Cross-sectional study | MBI and  Work Ability Index | 423 | High burnout: 6%  Insufficient work ability: 9.5-fold  higher if burnout exists | High burnout***:*** 7.1% (***Higher***). |
| **Wright** et al. | 2011 | Canada | Physicians at an academic health science center | Cross-sectional study | Copenhagen Burnout Inventory (CBI) | 210 | High work-related burnout :14% | High burnout***:*** 7.1% (***Lower***). |
| **Figueiredo-Ferraz** et al. | 2009 | Portugal | Education professionals | Cross-sectional study | “Spanish Burnout Inventory” in its Portuguese version for education professionals (CESQT-PE) | 211 | High burnout:14.2% | High burnout***:*** 7.1% (***Lower***). |
| **Rojas** et al. | 2009 | Colombia | Teachers in the National  Faculty of Public Health | Cross-sectional study | MBI-HSS | 89 | Medium burnout: 13.5%  Fair burnout: 22.5%  Extreme burnout: 19.1% | High burnout***:*** 7.1% (***Lower***). |
| **Soler** et al. | 2008 | 12 European Countries: Bulgaria, Croatia, France, Greece, Hungary, Italy, Malta Poland, Spain, Sweden, Turkey, United Kingdom | Family Physicians | Cross-sectional study | MBI-HSS | 1393 | High EE: 43%  High DP: 35.3%  Low PA: 68%  Median Sick Leave ≥ 3 days in the Last Year: 50.2% in high EE, 39.9% in high DP, 38.9% in low PA | High EE: 27.9% (***Lower***)  High DP: 18.3% (***Lower***)  High PA: 88.3% (***Higher***)  Moderate and high EE significantly predict high absenteeism rate (2.1 and 3.3 times the rates if low EE, respectively) |
